# Comparison of Insulin Glargine and Detemir Effects on Hormones of Appetite and Metabolic Control in Patients with Type 1 Diabetes: A Randomized Clinical Trial

**DOI:** 10.22037/ijpr.2021.114841.15059

**Published:** 2021

**Authors:** Maryam Razzaghy-Azar, Hosein Momeni, Mona Nourbakhsh, Mitra Nourbakhsh, Atefeh Talebi, Gholamreza Pourgholi, Fahimeh Zeinolabedinian

**Affiliations:** a *Aliasghar Children Hospital, Iran University of Medical Sciences, Tehran, Iran. *; b *Metabolic Disorders Research Center, Endocrinology and Metabolism Molecular-Cellular Sciences Institute, Tehran University of Medical Sciences, Tehran, Iran. *; c *Department of Biochemistry, School of Medicine, Iran University of Medical Sciences, Tehran, Iran. *; d *Colorectal Research Center, Iran University of Medical Sciences, Tehran, Iran.*

**Keywords:** Type1 diabetes, Agouti-related peptide, Leptin, Peptide-YY-3-36, Ghrelin, HbA1C, Insulin

## Abstract

The aim of this study was to compare the insulin glargine and detemir effects on hormons affecting appetite and metabolic control of patients with type 1 diabetes. This single-blind randomized clinical trial was conducted on patients aged 2 to 18 years with type 1 diabetes who were referred to the endocrinology department of Ali-Asghar Children Hospital in Tehran, from April to September 2019. Patients were randomly allocated to receive insulin Glargine or insulin Detemir. Before starting treatment, blood samples were obtained for routine biochemical tests and factors affecting appetite, including Leptin, Ghrelin, Aguti-Related Peptide (AGRP), and Peptide-YY3-36 (PYY 3-36). Patients were evaluated monthly and insulin dose was adjusted based on target glucose and carbohydrate counting. At the end of three months, the anthropometric values , HbA1C and factors that influence appetite were measured again in both groups, and the results were compared. A total of 40 children with a new onset of type 1 diabetes under 18 years who were hospitalized in Ali Asghar Children Hospital were randomly assigned into two groups as Glargine (n = 20) and Detemir (n = 20). The mean age of patients in the Glargine group was 11.07 ±4.18 years and in the Detemir group was 8.06 ± 3.56. In Glargine group HbA1C, Cholesterol, LDL, AGRP significantly decreased and leptin increased after treatment., while the change of BMI Z-score was not significant. There was a significant decrease of HbA1C in the Detemir group after treatment but there was no significant change of other variables. There was no significant difference for all the variables between two groups after treatment. There was no significant difference for BMI, metabolic control and appetite hormones between Glargine and Detemir groups. BMI-z score did not change in Glargine group while leptin increased and AGRP decreased after treatment. HbA1C decreased significantly after treatment in both groups.

## Introduction

Type 1 diabetes increases by 3 to 5% a year worldwide. Disease risks are influenced by a combination of immunological factors, genetic, and metabolic control (1, 2). Diabetes treatment is often challenging because it involves choosing an appropriate treatment regimen, setting a patient’s glycemic target, frequent blood glucose monitoring, and avoiding undesirable side effects. Using insulin is often associated with an increased risk of hypoglycemia and weight gain (3). Many patients experience weight gain following insulin therapy. Weight gain is a risk factor for cardiovascular disease (3-5). Currently, 50% of type 1 diabetic patients are overweight or obese, and they also have a higher waist to hip circumference ratio than healthy individuals (6).

Insulin Glargine is a recombinant human analog that produces a 24-hour effect in most but not all patients with type 1 diabetes (2). Insulin Detemir effect on blood glucose control is in the first 12 hours after injection, but with high dose injection, it has a longer effect (3).

Patients with type 1 and type 2 diabetes who receive Detemir insulin have less weight gain than those using NPH and glargine insulin (7). There may be several reasons for less weight gain in diabetic patients treated with detemir insulin, such as lower hypoglycemia (resulting in less eating to compensate for this complication), faster transfer of detemir from the blood-brain barrier (resulting in transmission of satiety signal to brain and inhibition of hepatic glucose production against peripheral glucose uptake) and increased adiponectin levels which is associated with weight loss and less eating (8-10).

The aim of this study was comparison of anthropometric values, some biochemical testes and hormonal factors affecting appetite in diabetic patients treated with detemir and glargine insulin. 

## Experimental

*Methods*

This single-blind randomized clinical trial was performed in Ali-Asghar Children Hospital in Tehran, the capital of Iran, from April to September 2019. Patients were included in the trial with the permission of their parents, and they complete the informed consent under the Declaration of Helsinki. The study was conducted after the ethics committee of Iran University of medical sciences approved it (IR.IUMS.FMD.REC.1398.052). 

According to the results of previous studies (11, 12), the effect size is obtained as 0.9, and with considering this result and the type 1 error as 0.05, the type 2 error as 0.2, the sample size for each group was obtained as 20. 

The study population was the patients aged 2 to 18 yr with newly diagnosed type 1 diabetes who were admitted in the Endocrinology ward of Ali-Asghar Children Hospital to start treatment with insulin and enrolled into the study. However, patients with these problems were excluded from the trial: (a) Side effects of treatment that did not allow follow-up; (b) history of diseases that can affect the results; (c) taking other medicines to control blood glucose. 

The eligible patients were assigned to two groups as insulin Glargine (n = 20) and insulin Detemir (n = 20), using the permuted blocked randomization. We used the block size of two, which had two permutations (G and D), “G” was used for “Glargine” and “D” for “Detemir”. We wrote “G” on one card and “D” on another card, and the first two patients received Glargine and Detemir respectively, and the second two patients received Detemir and Glargine, respectively, and this allocation was repeated until the sample size in each group was gained. This study was a single-blind trial, and patients did not know the nature of the medications. Furthermore, the trial groups were unknown for the statistical analyst, and analysis was conducted with artificial codes. 

The eligible patients were on regular insulin every 6 h for two days to evaluate the total daily insulin dose. then detemir and glargine were started for patients in each group. Before starting long-acting insulin, weight and height, were measured, and the appetite questionnaire was filled out. Fasting blood sampling for measurement of glucose and lipids were done. For evaluation of hormonal factors affecting appetite, 5 mL blood samples were collected in an EDTA tube and was immediately transferred to a refrigerated centrifuge at 4 °C, and the plasma was separated. Then, phenylmethyl sulfonic acid (PMSF) solution was added to the whole plasma (10 µL per mL of plasma). Plasma samples were divided between microtubes at the volume of 200 µL and 20 µL of HCl, and DPPI solution was added to the microtubes. All the microtubes were transferred to the freezer.

One of hormonal factors that we measured was ghrelin that is an acyl peptide with 28 amino acids. It is secreted mainly by the stomach, with strong effects on appetite stimulation. Ghrelin-Receptor is primarily found in neuropeptide-Y and agouti-related peptides (AGRP) in the hypothalamus, especially in the arcuate nucleus, which plays a key role in the regulation of appetite (13). Another hormone was leptin that is a 16 kDa hormone contains 167 amino acids and its circulating concentration is proportional to fat stores. It is secreted mainly by adipocytes, and can prevent fat accumulation and decreases appetite. Leptin binds to specific receptors in the hypothalamus and brainstem after passing the blood-brain barrier) (14), PEPTIDE-YY3-36 (PYY3-36) has a hormonal signal to control nutrient volume and is secreted by the intestinal L cells in response to nutrients. It binds to the Y2-receptor by crossing the blood-brain barrier in areas of the hypothalamus. Activation of this receptor causes a decrease in the level of neuropeptide-Y, which exerts its anorexic effect through a direct central effect as well as its effect on intestinal motility(15) and AGRP. AGRP is a human homologous protein that is a competitive endogenous antagonist of all melanocortin receptors (MCRs) (2). The long-acting insulin was adjusted as needed in 24 h, and the short-acting insulin was also calculated according to the carbohydrate counting. Blood glucose was measured by glucometer (Accu CHEK Performa) before and 2 h after the meal. The patients were visited free of charge by the doctor weekly for the first month and then monthly for 3 months. Their growth parameters, appetite status, and glucose control were measured in every visit. At the end of three months, hormones affecting appetite and the appetite and weight gain trend questionnaires were assessed again. 

 The paired *t*-test was used to compare continuous variables before and after intervention in the Detemir group and also in the Glargine group. The independent *t*-test was applied to compare mean differences between two groups before and after the intervention, and the chi-square or Fisher exact test is used to analyze qualitative variables. The Analysis of covariance (ANCOVA) was used to compare the mean difference between Detemir and Glargine groups by adjusting the confounder variables. Data analysis was conducted using SPSS software version 24. The significance level is considered as 0.05 in all statistical analyses. 

## Results

**Figure 1:**
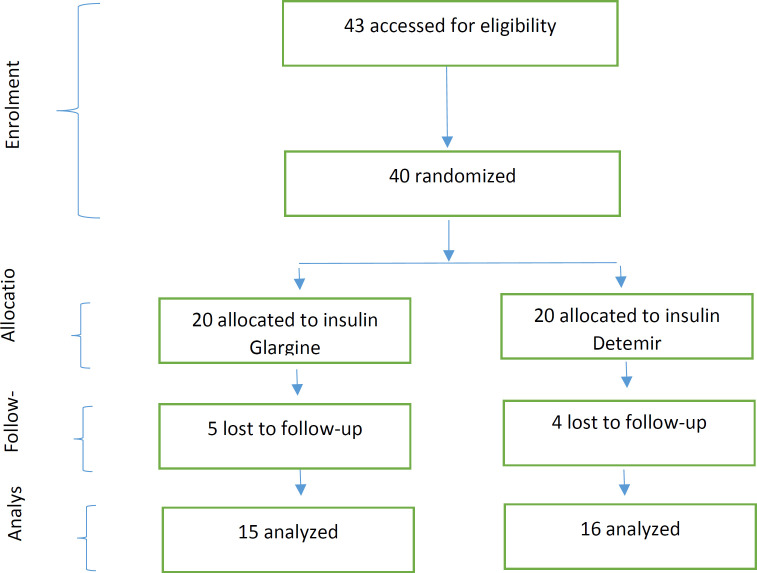
Flowchart of progress through the trial

Totally 40 eligible patients were identified, and all of them meted the inclusion criteria; and these patients were randomly allocated to two groups (insulin Glargine and insulin Detemir). Twenty of them were allocated to the insulin Glargine group, and 20 of them were allocated to the insulin Detemir group. Nine patients declined to follow up whom 5 patients belonged to the Glargine group, and 4 patients belonged to the Detemir group. The analysis was performed with 31 patients, of whom 15 patients were in Glargine and 16 patients in the Detemir group (Figure1). 

The demographic and baseline clinical and biochemical characteristics of the patients in the Glargine and Detemir groups are given in Table 1. The results show the statistically significant mean difference of age, height z-score, LDL, and cholesterol between two groups at baseline (Table 1). 

The results show that after the intervention, there is no statistically significant difference between two groups (*p* > 0.05). The analysis of age, height Z-Score, LDL, and cholesterol was conducted using analysis of covariance because their baseline values were significantly different between two groups (Table 2). 

Table 3 shows the mean and standard deviation (SD) of all the variables and *p* value of the paired t-test before and after treatment in each group that received insulin glargine or detemir. As shown in Table 3, HbA1C cholesterol, LDL, AGRP significantly decreased after treatment with glargine but leptin and hip circumference increased post-treatment (*P* < 0.05). While there was no significant difference between the mean of height, weight, and BMI z-score at pre- and post-treatment with this insulin (*P* > 0.05). For Detemir insulin, the mean of HbA1C was significantly better in post-treatment (Table 3) but there were no differences in other items. 

Also, the mean variables of child food intake and appetite questionnaire, emotional overeating ( EOE), enjoyment of food ( EF), desire to drink ( DD), satiety responsiveness ( SR), slowness in eating (SE), emotional under-eating (EUE), food fussiness (FF) were compared in the groups receiving insulin Glargine and Detemir at pre- and post-treatment, and there was no significant difference (*p* > 0.05). 

## Discussion

The aim of this study was to compare the effects of insulin Glargine and Detemir on the hormonal factors affecting appetite and psychological appetite factors in patients with type 1 diabetes. The results of the study showed that in the glargine group after intervention, HbA1C, cholesterol, LDL and AGRP significantly decreased. but leptin and hip circumference significantly increased. In Detemir group HbA1C significantly decreased after intervention but other variables did not change significantly

Davies *et al.* compared glycemic status, hypoglycemia, and weight changes over 26 weeks between insulin Detemir and NPH in patients with type 2 diabetes. Both groups had normal HbA1c glycosylation levels, and overweight was lower in patients taking insulin Detemir than in NPH patients, while hypoglycemia has been lower in patients treated by insulin Detemir; and the weight loss of the patients was attributed to insulin Detemir (16). In an interventional study, Dr. Montenana et al. investigated type I diabetic patients for 26 weeks and type 2 diabetic patients with a BMI of 25–40 kg/m2 treated with at least two insulin injections for more than 3 months. During this study, the weight gain and BMI of Detemir-treated patients were significantly lower than NPH during the 26 weeks, but the HbA1c changes were not statistically different between the two groups (17). In the study of Joseph R. Vasselli *et al.* on a mouse model, one dose of intracerebroventricular (icv) injection of insulin Detemir and regular insulin into the third ventricle of the brain was done. Then weight and energy consumption were compared between the two groups. In this study, low doses of both Detemir and regular insulin, were injected directly into the paraventricular nucleus of the brain of rats, reduced energy consumption, decreased appetite and weight loss over 48 and 120 h were higher in the detemir-treated group. In this study, ghrelin was used as a control that had an opposite effect on insulin (18). In the study of Paweł Olczyk *et al*., Leptin, its receptor, and adiponectin were compared in the blood of patients with type 2 diabetes before and after 6 months of insulin Detemir treatment. Insulin therapy normalized Leptin and adiponectin receptor levels in these patients, which had prevented insulin resistance and effectively controlled the appetite and weight of obese patients (19). In a study conducted by Jmrojas *et al*. on Sprague Dawley mice exposed to a 28-day fattening diet model, it was shown that insulin Detemir compared with insulin Glargine and NPH resulted in lower weight gain, especially in patients with higher BMI (20). In a study conducted by K.hermansen et al., the basal insulin Detemir along with short-acting insulin, and basal insulin NPH along with short-acting regular insulin in type 1 diabetic patients within 18 weeks were compared and found that there was less weight gain and hypoglycemia risk in patients receiving insulin Detemir along with insulin aspart compared with those who received NPH and regular insulin (21). In another study by LW van Golen *et al*., the insulin Detemir and NPH on appetite-regulating brain areas responded to dietary stimulation for 12 weeks in type 1 diabetic patients were compared and found that there was less brain activity in bilateral insula areas in patients who received insulin Detemir compared with NPH which is due to inhibition of brain activity in bilateral insula as an appetite regulator area in response to food stimuli (22).

**Table 1:** Baseline clinical characteristics of the glargine and Detemir groups

**Variable**	**Insulin Glargine**		**Insulin Detemir**		***p*** **-value**
	**mean**	**SD**	**mean**	**SD**	
Age	11.07	4.19	8.06	3.56	0.018^*^
Height Z-score	-1.07	2.19	0.39	0.90	0.017^*^
Weight Z-score	-1.84	4.09	0.12	0.73	0.117
BMI Z-score	-0.82	0.43	-0.14	0.96	0.441
Abdominal circumference	65.45	11.63	61.72	9.90	0.277
Hip circumference	72.74	13.20	67.67	10.08	0.175
FBS	167.09	56.97	169.80	77.00	0.899
Daily insulin dose	0.77	0.31	0.76	0.34	0.897
HBA1C	10.41	2.73	10.41	2.25	0.996
TG	119.24	68.17	114.00	48.27	0.779
cholesterol	189.14	50.99	146.75	19.28	0.001^*^
LDL	113.52	39.00	92.90	23.43	0.048^*^
HDL	45.71	10.64	36.65	17.88	0.054
AGRP	1.63	0.70	1.58	0.59	0.827
Leptin	1.06	0.57	1.19	0.54282	0.525
Ghrelin	0.40	0.35	0.49	0.2418	0.413
PYY3-36	1.12	0.65187	1.19	0.49843	0.742

^*^significance. AGRP, aguti-related peptide; PYY3-36, PEPTIDE-YY3-36.

**Table 2:** Comparison between the mean and SD of clinical and biochemical characteristics after intervention between the glargine and Detemir groups

**Variable**	**Insulin Glargine**		**Insulin Detemir**		***p*** **-value**
	**mean**	**SD**	**mean**	**SD**	
Age	11.33	4.18	8.29	3.55	0.017
Height Z-score	-1.16	2.14	0.67	1.19	0.53^b^
Weight Z-score	-2.02	4.36	0.11	0.79	0.399
BMI Z-score	-0.88	0.49	-0.52	1.42	0.254
Abdominal circumference	65.26	11.77	62.20	9.08	0.358
Hip circumference	73.93	13.19	70.02	11.53	0.32
FBS	154.14	72.07	155.60	61.34	0.945
Daily insulin dose	0.74	0.31	0.827	0.35	0.386
HBA1C	8.51	1.84	8.38	2.27	0.848
TG	102.76	57.85	97.20	39.02	0.721
cholesterol	155.95	37.00	138.20	21.39	0.068
LDL	98.09	30.58	83.62	22.01	0.091
HDL	44.62	11.35	38.80	10.71	0.1
AGRP	1.48	0.67	1.57	0.95	0.767
Leptin	1.35	0.70	1.32	0.81	0.9
Ghrelin	0.43	0.22	0.41	0.15	0.7
PYY3-36	1.22	0.79	1.33	0.71	0.679

*significance. AGRP, aguti-related peptide; PYY3-36, PEPTIDE-YY3-36

**Table 3:** Comparison of mean and SD before and after intervention in Glargine group and in Detemir ‎group

**Variable**	**Glargine Group**					**Detemir Group**				
	**before-Intervention**		**after- Intervention**			**before- Intervention**		**after- Intervention**		
	**Mean**	**SD**	**Mean**	**SD**	***P*** **-value**	**Mean**	**SD**	**Mean**	**SD**	***P-*** **value**
Height Z-score	-1.07	2.19	-1.16	2.14	0.455	0.39	0.90	0.67	1.19	0.053
Weight Z-score	-1.84	4.09	-2.02	4.36	0.31	0.12	0.73	0.11	0.79	0.918
BMI Z-score	-0.82	0.43	-0.88	0.49	0.721	-0.14	0.96	-0.52	1.42	0.094
Abdominal circumference	65.45	11.63	65.26	11.77	0.736	61.72	9.90	62.20	9.08	0.624
Hip circumference	72.74	13.20	73.93	13.19	0.048^*^	67.67	10.08	70.02	11.53	0.183
FBS	167.09	56.97	154.14	72.07	0.516	169.80	77.00	155.60	61.34	0.596
Total Daily insulin dose	0.77	0.31	0.74	0.31	0.421	0.76	0.34	0.827	0.35	0.289
HBA1C	10.41	2.73	8.51	1.84	0.013^*^	10.41	2.25	8.38	2.27	0.005^*^
TG	119.24	68.17	102.76	57.85	0.112	114.00	48.27	97.20	39.02	0.142
Cholesterol	189.14	50.99	155.95	37.00	0.006^*^	146.75	19.28	138.20	21.39	0.095
LDL	113.52	39.00	98.09	30.58	0.026^*^	92.90	23.43	83.62	22.01	0.054
HDL	45.71	10.64	44.62	11.35	0.664	36.65	17.88	38.80	10.71	0.66
AGRP	1.63	0.70	1.48	0.67	0.007^*^	1.58	0.59	1.57	0.95	0.959
Leptin	1.06	0.57	1.35	0.70	0.020^*^	1.19	0.54282	1.32	0.81	0.294
ghrelin	0.40	0.35	0.43	0.22	0.716	0.49	0.2418	0.41	0.15	0.25
PYY3-36	1.12	0.65187	1.22	0.79	0.41	1.19	0.49843	1.33	0.71	0.289

*significance. AGRP, aguti-related peptide; PYY3-36, PEPTIDE-YY3-36

## Conclusion

We concluded from this study HbA1C decreased after treatment with glargine and detemir. There was no significant difference on measured appetite factors between insulin Detemir and Glargine. Although Leptin increased and AGPR, LDL and Cholesterol decreased after treatment with glargine but there was no significant difference in the mean of height, weight, and BMI (Z-score) between the two groups and also between pre- and post-treatment in each group. 

## Funding

No funding to declare. 

## Conflict of Interest

The authors declare that they have no conflict of interest.

## Informed consent

Informed consent was obtained from parents before recruitment.

## Data availability

The datasets generated during the current study are available from the corresponding author on reasonable request.
